# Facile Preparation of Cobalt Nanoparticles Encapsulated Nitrogen-Doped Carbon Sponge for Efficient Oxygen Reduction Reaction

**DOI:** 10.3390/polym15030521

**Published:** 2023-01-19

**Authors:** Ying Leng, Kai Jin, Tian Wang, Hui Sun

**Affiliations:** 1 State Key Laboratory of High-Efficiency Coal Utilization and Green Chemical Engineering, School of Chemistry and Chemical Engineering, Ningxia University, Yinchuan 750021, China; 2 Department of Chemistry, University of Washington, Seattle, WA 98195, USA

**Keywords:** nitrogen-doped carbon sponge, oxygen reduction reaction, self-assembly, cobalt nanoparticle

## Abstract

The facile preparation of non-noble metal nanoparticle loaded carbon nanomaterials is promising for efficient oxygen reduction reaction (ORR) electrocatalysis. Herein, a facile preparation strategy is proposed to prepare nitrogen-doped carbon sponge loaded with fine cobalt nanoparticles by the direct pyrolysis of the cobalt ions adsorbed polymeric precursor. The polymeric sponge precursor with continuous framework and high porosity is formed by the self-assembly of a poly(amic acid). Taking advantage of the negatively charged surface and porous structure, cobalt ions can be efficiently adsorbed into the polymeric sponge. After pyrolysis, fine cobalt nanoparticles covered by carbon layers are formed, while the sponge-like structure of the precursor is also well-preserved in order to give cobalt nanoparticles loaded nitrogen-doped carbon sponges (Co/CoO@NCS) with a high loading content of 44%. The Co/CoO@NCS exhibits promising catalytic activity toward ORR with a half-wave potential of 0.830 V and a limiting current density of 4.71 mA cm^−2^. Overall, we propose a facile polymer self-assembly strategy to encapsulate transition metal nanoparticles with high loading content on a nitrogen-doped carbon sponge for efficient ORR catalysis.

## 1. Introduction

With the excessive consumption of fossil fuels, the energy crisis and environmental pollution are increasingly concerned [1]. The development of clean and renewable energy sources is imperative and of great significance [2]. As one of the most promising clean energies, fuel cells are highly efficient energy conversion devices that convert chemical energy directly into electricity [3,4,5,6]. However, due to the sluggish reaction kinetics of oxygen reduction reaction (ORR), the cathodic reaction of fuel cells, the rapid development of fuel cells is restricted. A large diversity of electrocatalysts was explored to promote the reaction kinetics and lower the activation energy barrier of ORR in order to improve the performance of fuel cells [7,8,9]. However, the most promising electrocatalyst toward ORR is so far still platinum (Pt)-based catalysts [7,8,9,10]. Considering the high cost, scarce reserves, and unsatisfied stability, etc., of Pt-based catalysts, the exploration of non-noble metal electrocatalysts is highly desired [10].

In the last few decades, carbon nanomaterials showed great potential as supports to load catalytic active species due to their excellent stability and extraordinary physicochemical properties [11,12,13,14,15,16]. Among the low dimensional carbon categories, including fullerene, carbon nanotubes, graphene, etc. [17], three dimensional porous carbon nanostructures showed advantages in mass and electron transfer due to the interconnected structure and high porosity [18,19]. As an alternative of Pt, transition metals, such as cobalt, copper, nickel, etc., and their single atoms, oxides, sulfides, phosphates, and hydroxides, etc., were widely encapsulated in porous carbon substrates for efficient ORR catalysis [20,21,22,23]. The doping of heteroatoms including nitrogen (N), sulfur (S), phosphorus (P), boron (B), and fluorine (F), etc., into carbon substrates is beneficial for the fixation of the active catalytic species and modulation of charge distribution [24,25,26], thus optimizing the adsorption and desorption process of active intermediates during electrocatalysis and promoting the electrocatalytic activity [27,28]. Nitrogen doping is the mostly studied and effective strategy to tailor the redistribution of the electron densities of the adjacent carbon atoms, which were proved to be the active sites for ORR [29,30,31]. However, the preparation of well-controlled small-sized metal nanoparticles encapsulated three dimensional porous carbon nanomaterials doped with heteroatoms is often accompanied with tedious procedures [32]. Typically, there are two steps, the fabrication of carbon supports and deposition of metal nanoparticles. The controlled preparation of three-dimensional porous carbon nanomaterials often relies on the sol-gel method and template-assisted strategy, etc. [33], which involves strenuous post-processing procedures [34,35]. Recently, the self-assembly of amphiphilic polymers was regarded as an emerging technology to prepare carbon nanomaterials with desired morphology and porosity, including nanospheres, sheets, nanobowls, fibers, and three-dimensional structures [36,37,38,39], demonstrating the versatility of polymer self-assembly to prepare functional nanomaterials [40,41,42,43].

**Figure 1:**
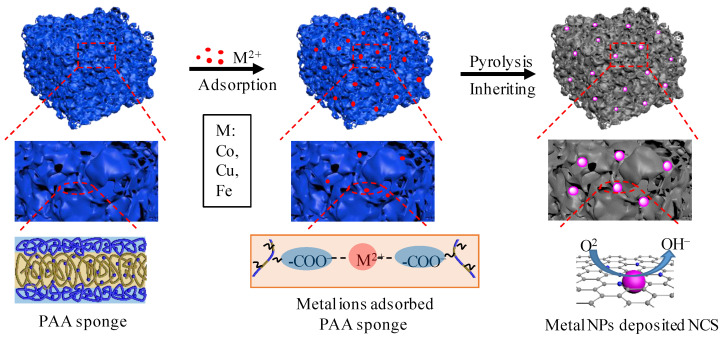
Schematic illustration of the preparation of M@NCS derived from the direct pyrolysis of metal ions that adsorbed PAA sponge for efficient ORR catalysis.

In this paper, we prepared fine cobalt nanoparticles encapsulated with nitrogen-doped carbon sponge (Co/CoO@NCS) by the direct pyrolysis of cobalt ions adsorbed polymeric precursors, as shown in Figure 1. The embedding of the nanoparticles into the framework of the carbon sponge and the coated thin carbon layer prevents the agglomeration of the nanoparticles. The amphiphilic poly(amic acid) (PAA) was prepared by the condensation polymerization of two commercially available monomers, pyromellitic dianhydride (PMDA) and 3,5-diamino-1,2,4-triazolz under mild conditions. Different metal ions (Co^2+^, Cu^2+^, and Co^2+^/Cu^2+^) can be efficiently adsorbed on the PAA sponge, taking advantage of the strong electrostatic interaction. Notably, the 3,5-diamino-1,2,4-triazolz has an ultrahigh nitrogen content of 70.7%, which guarantees the abundant nitrogen doping of the formed carbon sponge. Benefiting from the interconnected porous network structure, active nitrogen species and uniform distribution of small-sized cobalt nanoparticles, the Co/CoO@NCS exhibited favorable performance towards ORR.

## 2. Materials and Methods

### 2.1. Materials

Pyromellitic dianhydride (PMDA, 99%) and 3,5-diamino-1,2,4-triazolz (98%) were purchased from Aladdin Chemistry, Co., Ltd., Shanghai, China DMSO-*d*_6_ was purchased from J&K Scientific Ltd., Shanghai, China. *N, N*-Dimethylformamide (DMF, AR), acetone (AR), potassium hydroxide (KOH, 99.99%), cobalt nitrate hexahydrate (Co(NO_3_)_2_·6H_2_O, 98.5%), and copper nitrate hydrate (Cu(NO_3_)_2_·3H_2_O, 98.5%) were purchased from Sinopharm Chemical Reagent Co., Ltd., Shanghai, China. and used as received. Nafion solution (5 wt.%) was obtained from Sigma-Aldrich, Shanghai, China and Pt/C (20 wt.%) was purchased from Johnson Matthey, Shanghai, China.

### 2.2. Synthesis of Poly(amic acid) (PAA)

To synthesize PAA, PMDA (2.204 g, 10.00 mmol) was dispersed in 10 mL of DMF and 3,5-diamino-1,2,4-triazole (1.011 g, 10.00 mmol) was dissolved in 20 mL of DMF. Then the 3,5-diamino-1,2,4-triazole solution was placed in an ice bath, and the PMDA dispersion was added into the solution batch-by-batch in an hour (2 mL × 5 times). With the polymerization of the two monomers, the color of the mixture turned from gray to yellow. After stirring for 24 h at ambient temperature, the obtained stock solution was obtained and stored at −20 °C. Subsequently, the stock solution was precipitated in acetone three times to afford PAA powder for further characterizations.

### 2.3. Self-Assembly of PAA into Sponge

The PAA stock solution was diluted to 30 mg mL^−1^ by DMF (5 mL). Deionized water (*v*_water_:*v*_DMF_ = 2:1) was added dropwise to the PAA solution with a speed of 5 mL h^−1^ by a peristaltic pump under stirring (250 rpm). DMF was removed by dialysis of the solution in deionized water for 2 days. Then the PAA sponge was obtained by freeze-drying under vacuum.

### 2.4. Preparation of Co, Cu, and Co/Cu Nanoparticles-Loaded Nitrogen-Doped Carbon Sponge (M@NCS)

The Co, Cu, and Co/Cu nanoparticles-loaded nitrogen-doped carbon sponges were obtained by the direct pyrolysis of corresponding ions adsorbed PAA sponge. Specifically, 100 mg of PAA powder was re-dispersed in 10 mL of deionized water, and 66.6 mg of Co(NO_3_)_2_·6H_2_O, Cu(NO_3_)_2_·6H_2_O and the mixture of Co(NO_3_)_2_·6H_2_O and Cu(NO_3_)_2_·6H_2_O (*wt.*/*wt.* = 1:1) were added into the dispersion, respectively. Then the ions were allowed to be adsorbed into PAA sponge for 4 h. After centrifugation and being freeze-dried under vacuum, the ions adsorbed PAA sponges were obtained, which were then pyrolized at 800 °C for 1 h with a heating rate of 5 °C min^−1^ under nitrogen atmosphere to give different metal nanoparticles loaded nitrogen-doped carbon sponges (noted as Co/CoO@NCS, Cu@NCS, and Co/Cu@NCS).

### 2.5. Electrochemical Measurements

The electrochemical measurements were conducted by an PARSTAT 4000 electrochemical workstation (AMETEK, San Diego, CA, USA) equipped with PINE rotary disk electrode (RDE 5 mm), using a traditional three-electrode system. A glassy carbon working electrode (GCE, 5 mm inner diameter, 0.196 cm^2^), a graphite rod as a counter electrode, and a Hg/HgO reference electrode were used for all the tests. The working electrode was prepared as follows: 2 mg of the samples was dispersed in the mixture of 300 μL of ultra-pure water, 100 μL of isopropanol, and 5 μL of Nafion (5 wt.%) under ultrasonication for 30 min. Then, the catalyst ink (5 μL) was dropped onto the glassy carbon working electrode four times, giving a total volume of 20 μL and loading content of 0.50 mg cm^−2^. The preparation of commercial Pt/C working electrodes is the same as above procedures. For the oxygen reduction reaction (ORR) test, the electrolyte (0.1 M KOH aqueous solution) was saturated with N_2_ and O_2_ for 30 min, respectively. Cyclic voltammetry (CV) curves of the catalysts were typically measured in the potential range of −0.8–0.3 V (vs. RHE) at a scan rate of 50 mV s^−1^ in N_2_-saturated or O_2_-saturated 0.1 M KOH solutions, respectively. Linear sweep voltammetry (LSV) curves were obtained at a scan rate of 5 mV s^−1^ with a rotating speed from 400 to 2500 rpm. The electrochemical impedance spectra (EIS) were recorded with an amplitude of 5 mV and a frequency from 0.01 Hz to 100 kHz. The rotating ring-disk electrode (RRDE) test was performed to determine the electron transfer numbers (n), which were also confirmed by the Koutecky–Levich (K–L) equation. The equation is as follows:

1/*J* = 1/*J*_K_ + 1/*J*_L_ = 1/*J*_K_ + 1/(B*ω*^1/2^) (1)

B = 0.62nFC_0_(D_0_)^2/3^*v*^−1/6^ (2)

*J*_K_ = nF*k*C_0_ (3)

where *J* is the measured current density (mA cm^−2^), *J*_L_ and *J*_K_ are the limiting and kinetic current density (mA cm^−2^), *ω* is the angular velocity of the rotating disk electrode in rad s^−1^, F is the Faraday constant (96,485 C mol^−1^), C_0_ (1.2 × 10^−6^ mol cm^−3^) and D_0_ (1.9 × 10^−5^ cm^2^ s^−1^) are the volume concentration and diffusion coefficient of O_2_ saturated 0.1 M KOH electrolyte, *v* (0.01 cm^2^ s^−1^) is the viscosity of the electrolyte, and *k* is the electron transfer rate constant.

### 2.6. Characterizations

Fourier transform infrared spectroscopy (FTIR) analyses were carried out with a TENSOR II (Bruker, Karlsruhe, Germany), and the proton nuclear magnetic resonance (^1^H NMR) spectrum was recorded using a Bruker AVANCE III 400 MHz spectrometer (Bruker, Karlsruhe, Germany) with DMSO-*d*_6_ as solvent. AGILENT 1260II gel permeation chromatography (GPC) (AGILENT, Santa Clara, CA, USA) was used to quantify the relative molecular weight and molecular weight distribution of PAA at 40 °C with DMF as eluent at 0.8 mL min^−1^. The nitrogen adsorption/desorption experiment operated with Autosorb iQ (Anton Paar, Graz, Austria) was conducted to determine the specific surface area and pore distribution of Co/CoO@NCS, Cu@NCS, and Co/Cu@NCS. The specific surface areas of the samples were calculated by the Brunauer–Emmett–Teller (BET) method in the relative pressure (P/P_0_) range of 0.05 to 0.35, and the pore size distribution was calculated using the Barrett–Joyner–Halanda (BJH) model. X-ray diffraction (XRD) was conducted on a Smartlab powder diffractometer (Rigaku, Tokyo, Japan) operated at 40 kV with a scan rate of 5° min^−1^ from 10° to 85°. The Raman spectra of Co/CoO@NCS, Cu@NCS, and Co/Cu@NCS were recorded using a Thermo Fisher DXR Raman spectrometer (Thermo Fisher, Waltham, MA, USA). The micromorphology of the samples was observed by a Hitachi Regulus 8100 cold field emission scanning electron microscope (SEM) (Hitachi, Tokyo, Japan) at 5 kV and 10 μA. Transmission electron microscope (TEM) images were obtained by using Hitachi HT7700 (Hitachi, Tokyo, Japan) with an acceleration voltage of 100 kV. High-resolution TEM images, high-angle annular dark field scanning TEM (HAADF-STEM) images, and elemental mappings were recorded by a FEI TECNAI G2 F20 electron microscope (FEI, Hillsboro, OR, USA) operated at 200 kV. X-ray photoelectron spectroscopy (XPS) spectra were obtained by using Thermo SCIENTIFIC ESCALAB 250Xi (Thermo Fisher, Waltham, MA, USA) under vacuum.

## 3. Results and Discussion

### 3.1. Synthesis and Self-Assembly of PAA

The PAA is synthesized by the condensation polymerization of PMDA and a high-nitrogen-containing diamine (70.7%, 3,5-diamino-1,2,4-triazole) under mild condition, as shown in Figure S1. Fourier transform infrared spectroscopy (FTIR) and proton nuclear magnetic resonance (^1^H NMR) were performed to confirm the successful synthesis of the PAA (Figures S2 and S3). To induce the self-assembly of PAA, the raw solution of PAA was diluted to a concentration of 30 mg mL^−1^ followed by the direct addition of deionized water dropwise under stirring. After dialysis against water to remove the organic solvent, the morphology of the assemblies was observed by scanning electron microscope (SEM) and transmission electron microscope (TEM), demonstrating a sponge-like structure with high porosity (denoted as PAA sponge), as illustrated in Figure S4. It should be noted that the PAA sponge has an interconnected framework formed by the hydrophobic backbone of the PAA (Figure 1), and abundant mesopores ranging from several to tens of nanometers. The aqueous solution of PAA sponge was freeze-dried under vacuum to give powders for the following procedures.

### 3.2. Preparation and Characterization of M@NCS

Taking advantage of the hydrophilicity and negatively charged characteristic of the carboxylic group in the PAA, cationic metal ions can be effectively adsorbed into the hydrophilic part of PAA sponge by electrostatic interaction (Figure 1). Therefore, we re-dispersed the PAA sponge powder in water, followed by the addition of metal ions, including Co^2+^, Cu^2+^, and Co^2+^/Cu^2+^. After adsorption of corresponding metal ions, the PAA sponge was precipitated at the bottom of the beaker due to the neutralization of negative charges, and the morphological features of the PAA sponge were maintained after the adsorption of Co^2+^, as shown in Figure S5. The metal ions adsorbed PAA sponge were centrifuged and washed with water to remove free ions. With the pyrolysis of the metal ions adsorbed PAA sponge at nitrogen atmosphere at 800 °C for 1 h, the corresponding metal (oxide) nanoparticles encapsulated by nitrogen-doped carbon sponges were obtained, denoted as Co/CoO@NCS, Cu@NCS, and Co/Cu@NCS, respectively. During pyrolysis, the PAA underwent an intramolecular cyclization reaction to form polyimide and then be converted to carbon (Figure S1). Hence, the sponge-like structure of the PAA sponge can be well-inherited because of the excellent thermal stability of polyimide. The thermogravimetric (TG) curve in Figure S6 recorded the change in mass percentage with temperature of Co^2+^ adsorbed PAA sponge, indicating a yield of Co/CoO@NCS of 22%.

**Figure 2:**
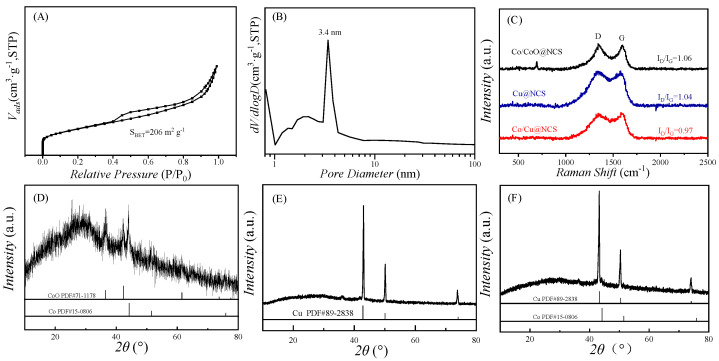
(**A**,**B**) Nitrogen adsorption–desorption isotherm and pore distribution curve of Co/CoO@NCS, (**C**) Raman spectra, and (**D**–**F**) XRD patterns of Co/CoO@NCS, Cu@NCS, and Co/Cu@NCS.

The specific surface areas and pore distributions of M@NCS were confirmed by nitrogen adsorption/desorption experiments, as shown in Figure 2A,B and Figure S7. The adsorption/desorption isotherm in Figure 2A indicates a specific surface area of 206 m^2^ g^−1^ of Co/CoO@NCS and the pore size mainly distributed at 3.4 nm (Figure 2B). For Cu@NCS and Co/Cu@NCS, the specific surface areas are comparable with that of Co/CoO@NCS (232 and 207 m^2^ g^−1^, respectively, Figure S7A,C). However, the pore distributions of Cu@NCS and Co/Cu@NCS are different from that of Co/CoO@NCS. Micropores with small-sized mesopores are observed for Cu@NCS (Figure S7B) and pores with a broad range of 0.8 nm to more than 10 nm are found for Co/Cu@NCS (Figure S7D). Figure 2C shows the Raman spectra of Co/CoO@NCS, Cu@NCS, and Co/Cu@NCS. The spectra show a typical D band at 1340 cm^−1^ and a G band at 1575 cm^−1^, which can be ascribed to the carbon lattice defects and degree of graphitization order in carbon materials. The intensity ratio of D band and G band (I_D_/I_G_) are commonly used to determine the degree of graphitization of carbon materials. The I_D_/I_G_ values of Co/CoO@NCS, Cu@NCS, and Co/Cu@NCS are 1.06, 1.04, and 0.97, respectively, indicating a high degree of graphitization of M@NCS due to the catalytic effect of transition metal ions during pyrolysis. The X-ray diffraction (XRD) patterns of Co/CoO@NCS, Cu@NCS, and Co/Cu@NCS are shown in Figure 2D–F. The diffraction peaks at around 44.28° and 51.7° in Figure 2D can be assigned to the (002) and (111) planes of Co, while the diffraction peaks centered at 36.35°, 42.47°, and 61.55° are ascribed to (111), (200), and (220) planes of CoO, indicating the coexistence of Co and CoO species. In addition, the relatively low diffraction intensity also implies the small diameter of Co and CoO nanoparticles. On the contrary, the strong diffraction peak at 43.29°confirms the formation of large-sized Cu nanoparticles (Figure 2E). When co-loading Co and Cu, the diffraction peaks of Cu are dominant and the diffraction peaks of Co are barely observed, demonstrating that the adsorption of Cu^2+^ is more favorable than that of Co^2+^ by PAA sponge. The inductively coupled plasma optical emission spectrometer (ICP-OES) results also prove the speculation, which show a loading content of 44.3 wt.%_Co_ for Co/CoO@NCS, 33.8 wt.%_Cu_ for Cu@NCS, and 5.45 wt.%_Co_/25.06 wt.%_Cu_ for Co/Cu@NCS, respectively.

**Figure 3:**
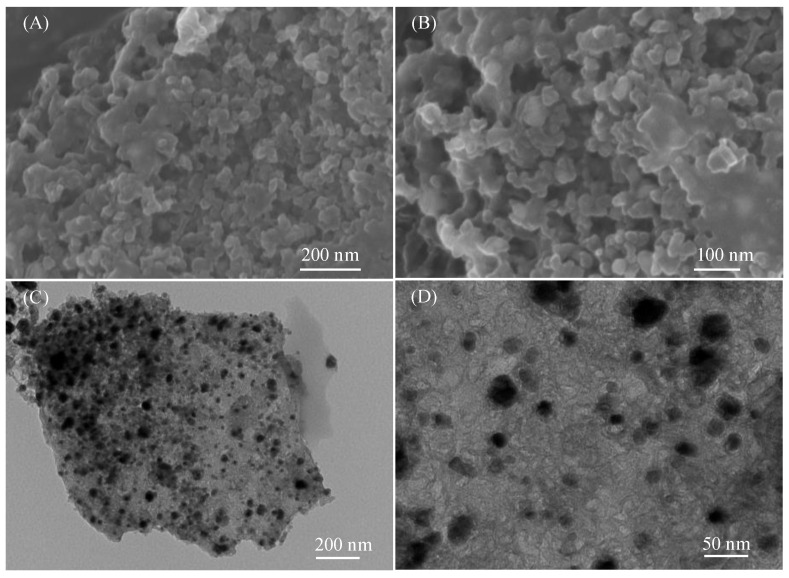
(**A**,**B**) SEM images and, (**C**,**D**) TEM images of Co/CoO@NCS.

The morphological features of the M@NCS were revealed by SEM and TEM, as shown in Figure 3 and Figure S8. It is clearly observed from the SEM and TEM images of Co/CoO@NCS in Figure 3A–D that the sponge-like structure of the precursor is well-preserved after pyrolysis. In addition, small-sized Co/CoO nanoparticles ranging from several to tens of nanometers are encapsulated in the framework of the carbon sponge, and a large number of mesopores also coexist. The carbon layers coated on the nanoparticles prevent the agglomeration of the Co/CoO nanoparticles. However, the particle sizes of Cu@NCS and Co/Cu@NCS dramatically increase to more than 100 nanometers with the same preparation procedure and conditions, as illustrated in the SEM and TEM images in Figure S8A–F. We speculate that the small particles of Cu and Cu/Co aggregate to form large sized particles during pyrolysis. The melting points for Cu and Co are 1083 and 1459 °C, respectively, which leads to the molten state and agglomeration of Cu nanoparticles other than Co/CoO nanoparticles at high temperature (800 °C) due to nano-size effect.

**Figure 4:**
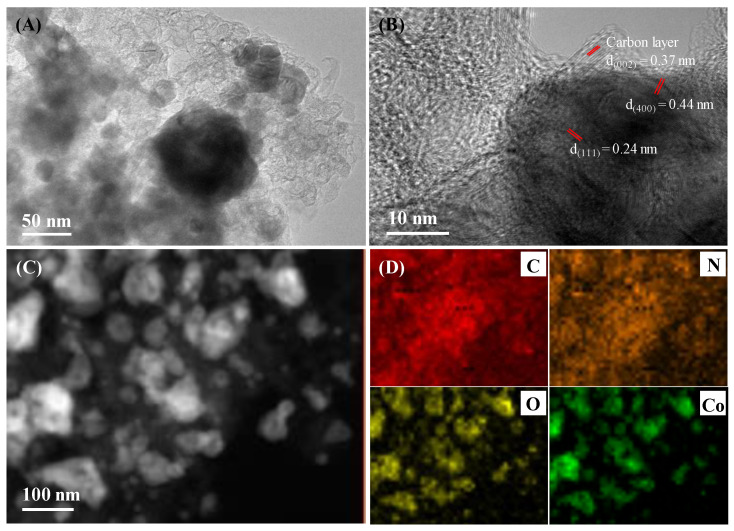
(**A**,**B**) HR-TEM images, (**C**) HAADF-STEM image, and (**D**) corresponding element mappings of Co/CoO@NCS.

High-resolution TEM (HR-TEM), high angle annular dark field scanning TEM (HAADF-STEM), and elemental mapping were performed to reveal the fine structure and elemental distribution of Co/CoO@NCS, as shown in Figure 4. The HR-TEM images in Figure 4A,B indicate the uniform distribution of Co/CoO nanoparticles on the carbon sponge. The nanoparticles are wrapped by graphitic carbon layers. The interplanar spacing of the (002) plane of graphitic carbon is measured to be 0.37 nm, and the lattice fringe with a distance of 0.24 and 0.44 nm is ascribed to the (111) plane of CoO and (400) plane of Co, respectively, indicating the coexistence of Co and CoO nanoparticles. Furthermore, the HAADF-STEM image and elemental mapping in Figure 4C,D verifies the homogeneous distribution and overlapping of oxygen and Co, further confirming the formation of CoO nanoparticles.

**Figure 5:**
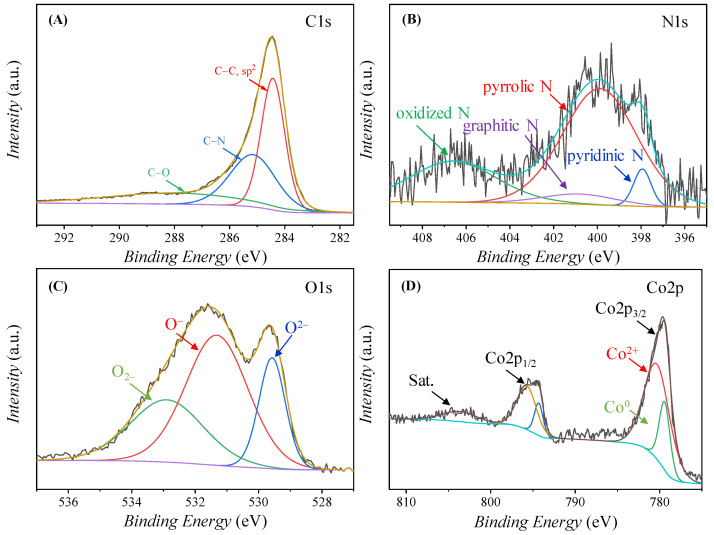
High-resolution XPS spectra of Co/CoO@NCS, (**A**) C1s, (**B**) N1s, (**C**) O1s, and (**D**) Co2p.

In order to analyze the composition and chemical microenvironment of the elements of Co/CoO@NCS, Cu@NCS, and Co/Cu@NCS, X-ray photoelectron spectroscopy (XPS) tests were carried out. Figure S9 shows a full survey of the surface elemental composition of Co/CoO@NCS, demonstrating the existence of carbon, nitrogen, oxygen, and cobalt, while the fine spectra of C1s, N1s, O1s, and Co2p in Figure 5A–D give detailed information on the corresponding elements. The high-resolution C1s spectrum (Figure 5A) can be divided into three peaks centered at 284.8, 285.2, and 288.0 eV, corresponding to the sp^2^ carbon, C−N, and C−O bond. There are four peaks that can be fitted in the N1s spectrum in Figure 5B, which can be assigned to pyridine N (397.9 eV), pyrrole N (399.9 eV), graphitic N (400.8 eV), and oxidized N (406.6 eV), respectively [44,45,46]. The O1s spectrum (Figure 5C) can be subdivided into three peaks, located at 529.6 eV for lattice oxygen, 531.3 eV for oxygen vacancies, and 532.9 eV for adsorbed oxygen, respectively. Furthermore, two peaks are fitted in the Co2p_3/2_ spectrum (Figure 5D), corresponding to Co^0^ (779.4 eV) and Co^2+^ (780.3 eV), indicating the formation of Co and CoO nanoparticles [47,48]. In addition, we also performed XPS analysis of Cu@NCS and Co/Cu@NCS, as shown in Figures S10–S12. The survey spectra in Figure S10 of Cu@NCS and Figure S12A of Co/Cu@NCS confirm the existence of carbon, nitrogen, oxygen, cobalt, and copper. The analysis results of the high resolution C1s and N1s spectra of Cu@NCS and Co/Cu@NCS are similar with that of Co/CoO@NCS, confirming the excellent stability of carbon sponge for encapsulation of different species. However, the fine O1s spectra of Cu@NCS and Co/Cu@NCS are obviously changed (Figures S11C and S12D). The disappearance of the lattice oxygen at lower binding energy demonstrates that the metals are in an unoxidized state, and the Cu2p spectra in Figures S11D and S12F and Co2p spectrum in Figure S12E further confirm the conclusion.

### 3.3. Electrocatalytic Performance toward ORR

**Figure 6:**
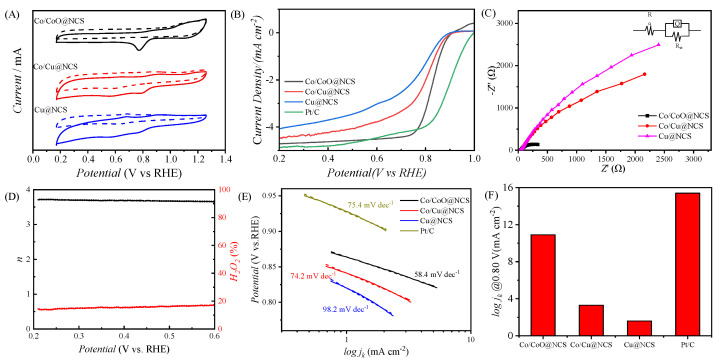
Electrocatalytic activity of M@NCSs for ORR: (**A**) CV curves in nitrogen saturated and oxygen saturated electrolyte, (**B**) LSV curves of M@NCSs and Pt/C, (**C**) EIS spectra of M@NCS, (**D**) electron transfer number and hydrogen peroxide yield of Co/CoO@NCS measured by RRDE, (**E**) Tafel plots, and (**F**) summary of the kinetic current densities of M@NCS and Pt/C at 0.80 V.

In our previous study [49], we investigated the ORR catalytic performance of NCS, exhibiting an onset potential (E_onset_) of 0.940 V vs. RHE, a half-wave potential (E_1/2_) of 0.816 V vs. RHE, and a limiting current density (J_L_) of 4.34 mA cm^−2^. In order to further improve the catalytic activity, we used NCS as supports to load various transition metal nanoparticles. The ORR catalytic activity of the as-prepared samples was evaluated in 0.1 M KOH aqueous solution at room temperature. Linear sweep voltammetry (LSV) and cyclic voltammetry (CV) tests were carried out in a three-electrode system by using a rotating disk electrode (RDE) as a working electrode. Figure 6A shows the CV curves of Co/CoO@NCS, Co/Cu@NCS, and Cu@NCS in N_2_-saturated and O_2_-saturated electrolytes at a scanning speed of 50 mV s^−1^. Compared with N_2_-saturated electrolyte, obvious oxygen reduction peaks appear in O_2_-saturated electrolyte. The reduction peak of Co/CoO@NCS locates a potential of 0.78 V, which is higher than those of Cu@NCS and Co/Cu@NCS. The more positive peak potential usually indicates the better catalytic activity for ORR. LSV curves in Figure 6B were measured in O_2_-saturated electrolyte with a rotating speed of 1600 rpm. The Co/CoO@NCS exhibits a E_onset_ of 0.950 V vs. RHE, a E_1/2_ of 0.830 V vs. RHE, and a J_L_ of 4.71 mA cm^−2^, which is comparable to Pt/C (E_onset_ of 0.990 V vs. RHE, E_1/2_ of 0.863 V vs. RHE, and J_L_ of 4.87 mA cm^−2^) and much better than that of Cu@NCS and Co/Cu@NCS, as well as the NCS. We speculated that the synergistic effect between NCS and Co/CoO nanoparticles, e.g., the interaction between Co/CoO nanoparticles and the active nitrogen species might be responsible for the improvement of ORR catalytic activity. Figure 6C shows the Nyquist plots of Co/CoO@NCS, Cu@NCS, and Co/Cu@NCS. The semicircular diameter of the electrochemical impedance spectra (EIS) of Co/CoO@NCS is much smaller than that of Cu@NCS and Co/Cu@NCS. The charge transfer resistance of Co/CoO@NCS is measured to be 90.9 Ω, which is much smaller than that of Cu@NCS (1777 Ω) and Co/Cu@NCS (737.1 Ω), indicating the fastest reaction kinetics of Co/CoO@NCS for ORR. To further estimate the ORR reaction kinetics of Co/CoO@NCS, LSV tests at a rotation speed of 400 to 2500 rpm were carried out (Figure S13). Based on the LSV curves at different rotation speeds, the Koutecky–Levich diagram and the electron transfer number (n) were obtained according to the K–L equation. The n was calculated to be 3.55–3.67 at a potential range of 0.2–0.6 V vs. RHE, indicating the quasi 4 electron transfer process of Co/CoO@NCS. The n and the yield of H_2_O_2_ during ORR process were also measured via rotating ring-disk electrode (RRDE), showing an n of ~3.70 and a H_2_O_2_ yield of 14–17% (Figure 6D), which is consistent with that calculated from the K–L equation.

The Tafel slope curves of M@NCS and Pt/C are shown in Figure 6E. The Co/CoO@NCS had the smallest slope of 58.4 mV dec^−1^, demonstrating the fastest reaction kinetics, while the Cu@NCS, Co/Cu@NCS, and Pt/C deliver large slopes of 98.2 mV dec^−1^, 74.2 mV dec^−1^, and 75.4 mV dec^−1^. Subsequently, the kinetic current densities of M@NCS and Pt/C at 0.80 V vs. RHE were also calculated, as illustrated in Figure 6F. The Co/CoO@NCS exhibits a current density of 10.9 mA cm^−2^, which is smaller than that of Pt/C (15.4 mA cm^−2^) but much higher than that of Cu@NCS (1.6 mA cm^−2^) and Co/Cu@NCS (3.3 mA cm^−2^). We speculate that the reason for the best performance of Co/CoO@NCS among M@NCS is ascribed to the fine particle size and formation of CoO. Electrochemical active area and surface roughness of Co/CoO@NCS were further analyzed by electrochemical double-layer capacitance (C_dl_) to evaluate the exposure of active sites and enhanced catalytic activity. It can be seen from Figure S14 that the C_dl_ of Co/CoO@NCS is 34.4 mF cm^−2^, corresponding to an electrochemcial active surface area (ECSA) of 172 m^2^ g^−1^, indicating that Co/CoO@NCS can provide abundant active sites for ORR, which is much higher than that of Cu@NCS and Co/Cu@NCS (~20 mF cm^−2^). In order to verify the stability of Co/CoO@NCS, the LSV curve after 1000 CV cycles was investigated, as presented in Figure S15. The E_1/2_ of Co/CoO@NCS is 0.800 V vs. RHE after 1000 cycles with an attenuation of 30 mV, while the E_1/2_ of Pt/C decays to 0.780 V vs. RHE, indicating the better cycle stability of Co/CoO@NCS. We speculated that the good catalytic activity and stability of Co/CoO@NCS was ascribed to several reasons: (1) the high porous structure of NCS with ultrathin frameworks, which facilitated the charge and mass transfer [49]; (2) the small size of Co/CoO nanoparticles; and (3) the synergistic effect between NCS and ultrafine Co/CoO nanoparticles.

## 4. Conclusions

In conclusion, we propose a facile polymer self-assembly strategy to prepare fine cobalt nanoparticle encapsulated nitrogen-doped carbon sponge for efficient ORR electrocatalysis. The PAA sponge with high porosity is fabricated by the self-assembly of an amphiphilic PAA. Taking advantage of the hydrophilicity and negatively charged surface of the sponge, metal ions can be effectively adsorbed, which converts to fine nanoparticles encapsulated carbon sponge by pyrolysis. The interconnected channels, efficient doping of nitrogen, as well as the small size of the Co/CoO nanoparticles, promote the exposure of active sites for electrocatalytic ORR. The Co/CoO@NCS exhibits decent electrocatalytic activity for ORR in alkaline media with an onset potential of 0.950 V vs. RHE and a half-wave potential of 0.830 V vs. RHE, which is comparable to that of Pt/C. Considering the large amount varieties of PAA and their versatile self-assembly behaviors, carbon nanomaterials with desired structures and chemical compositions can be efficiently prepared and derived from the pyrolysis of PAA precursors to establish a platform for loading of catalytic active species.

## Data Availability

The data can be accessed from the corresponding author.

## Supplementary Materials

The following supporting information can be downloaded at: https://www.mdpi.com/article/10.3390/polym15030521/s1, Figures S1–S15.
