# Supplementary material for: Facile Preparation of Cobalt Nanoparticles Encapsulated Nitrogen-Doped Carbon Sponge for Efficient Oxygen Reduction Reaction
Source: Polymers (Basel). 2023 Jan 19;15(3):521. doi: 10.3390/polym15030521 (PMC9920104; doi:10.3390/polym15030521)
Supplement: Supplementary file 1 [file polymers-15-00521-s001.zip › polymers-2172118-supplementary.pdf]

Supporting Information for

Facile Preparation of Cobalt Nanoparticles Encapsulated Nitrogen-Doped Carbon Sponge for Efficient Oxygen Reduction Reaction

Ying Leng [1], Kai Jin [1], Tian Wang [2] and Hui Sun [1,*]

[1]School of Chemistry and Chemical Engineering, State Key Laboratory of High-Efficiency Coal Utilization and Green Chemical Engineering, Ningxia University, Yinchuan 750021, China E-mail: sunhui@nxu.edu.cn

[2]Department of Chemistry, University of Washington, Seattle, WA 98195, United States.

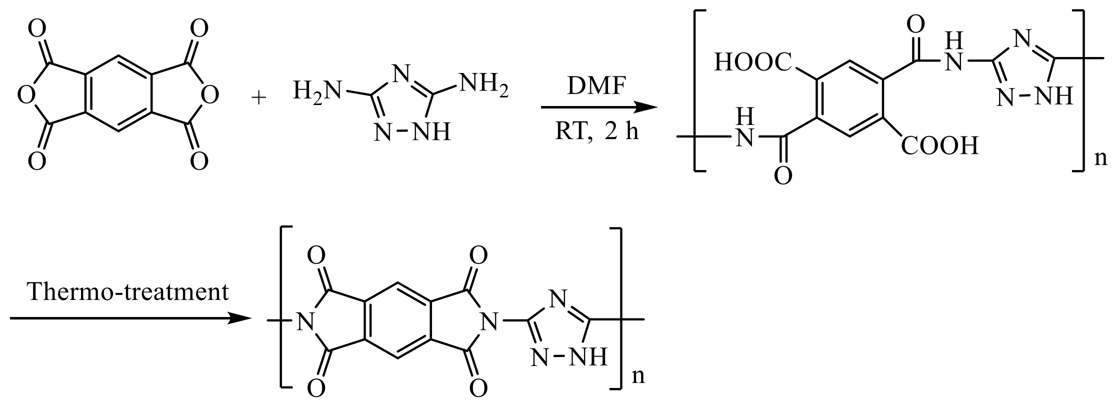

Figure S1. Synthesis and intramolecular imidization reaction of PAA.

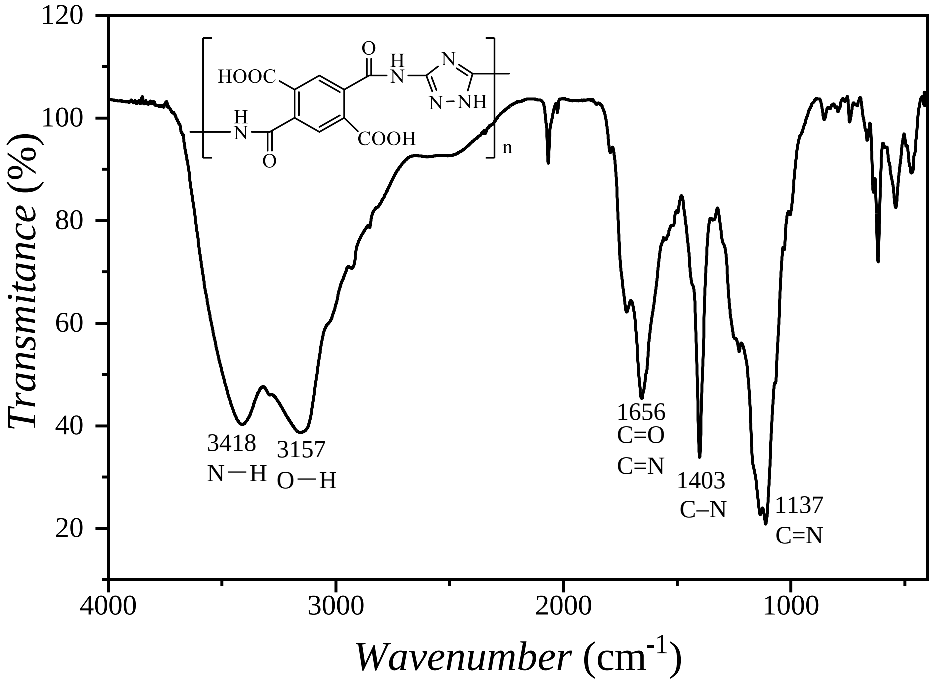

Figure S2. FTIR spectrum of PAA.

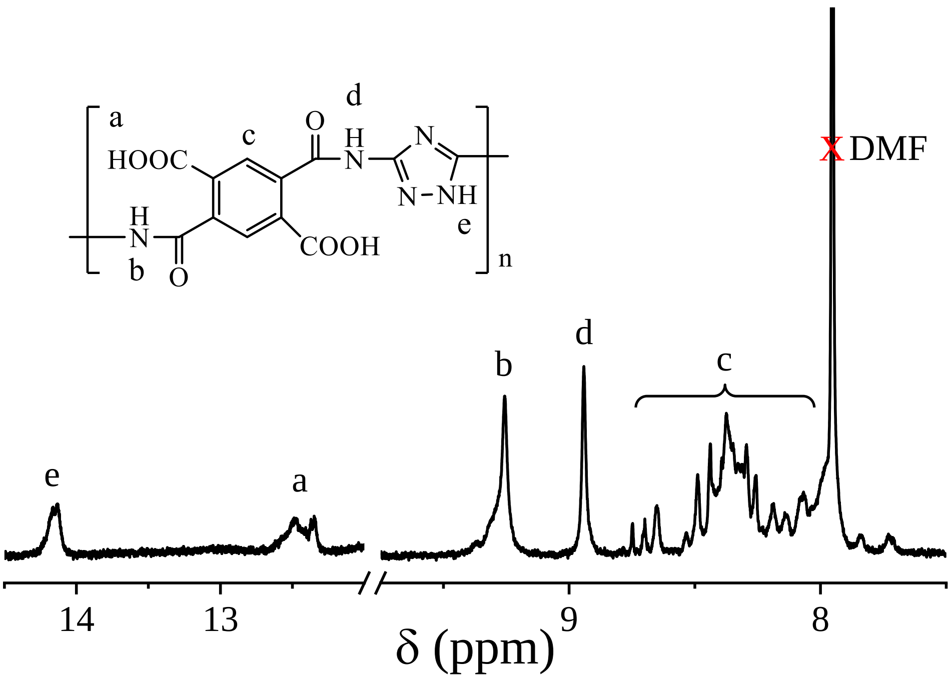

Figure S3. ^{1}H NMR spectrum of PAA in DMSO-d_6.

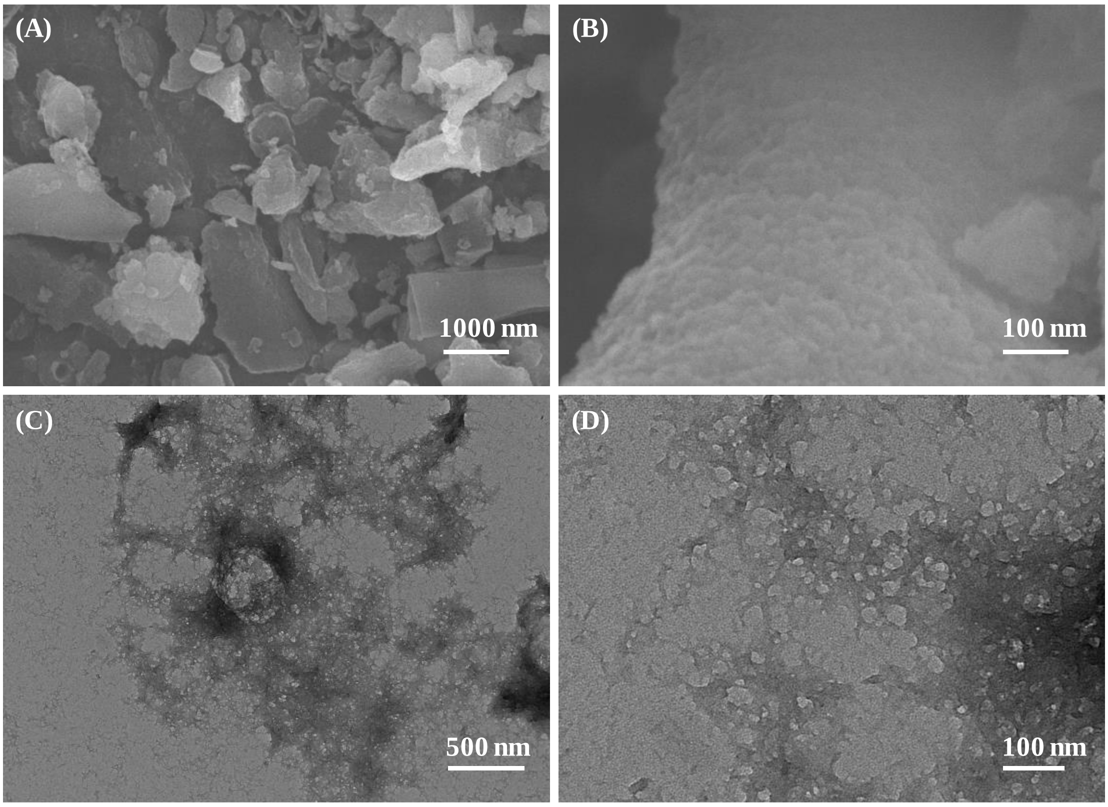

Figure S4. (A, B) SEM and (C, D) TEM images of PAA sponge.

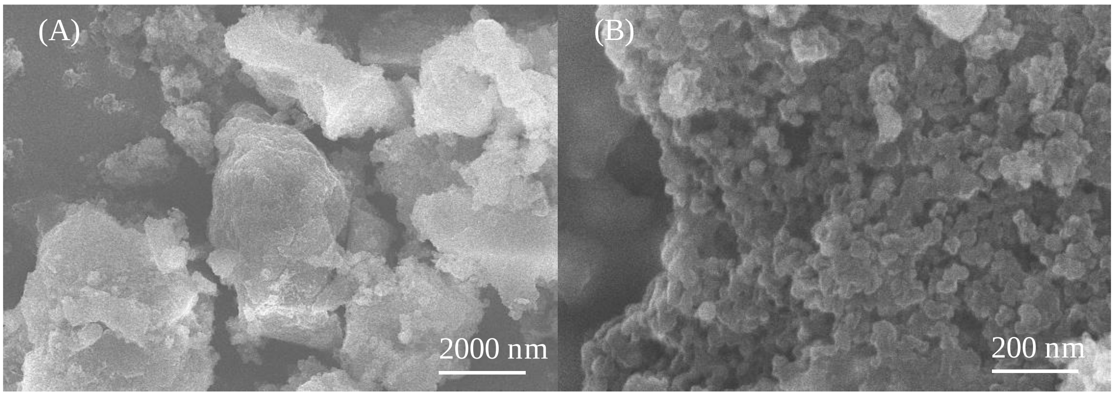

Figure S5. (A) and (B) SEM images of Co^{2+} adsorbed PAA sponge at different magnifications.

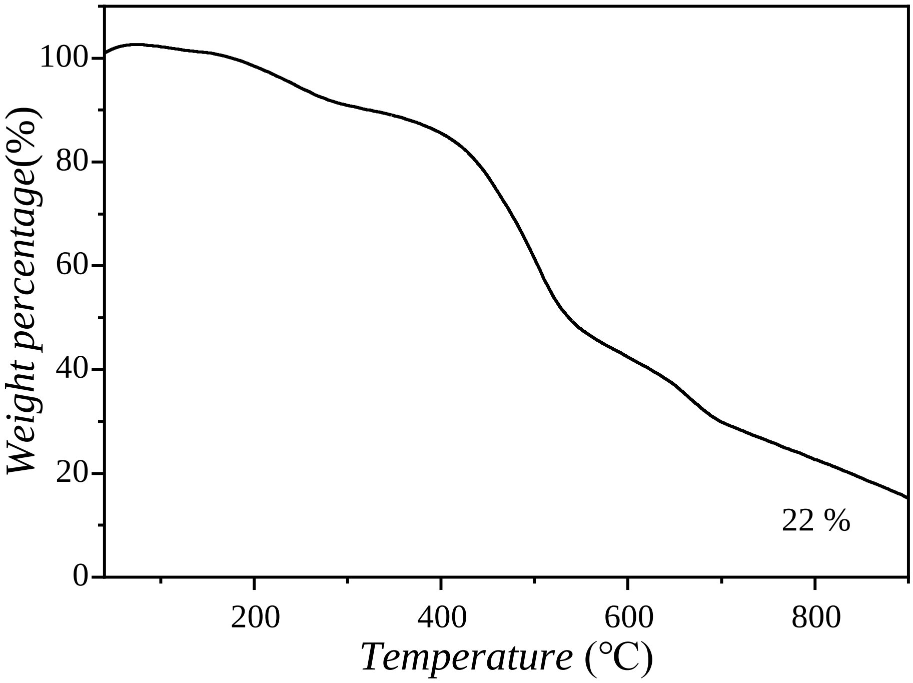

Figure S6. TG curve of Co/CoO@NCS at nitrogen atmosphere.

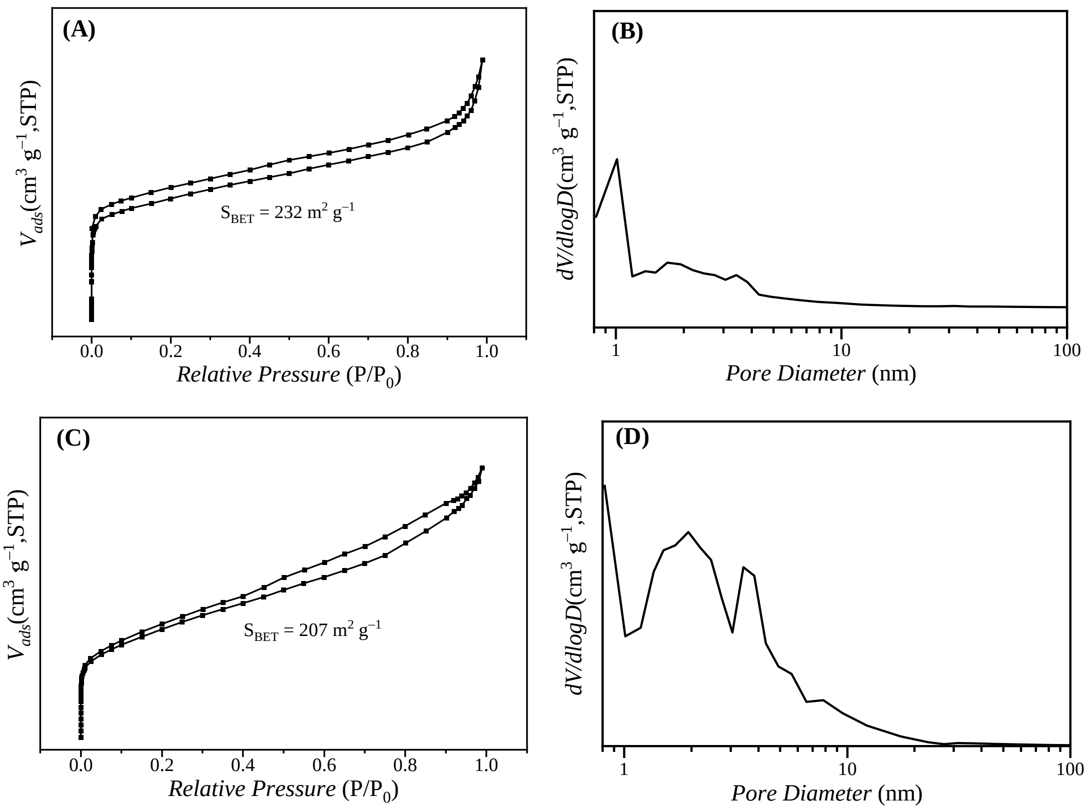

Figure S7. Nitrogen adsorption/desorption isotherm and pore size distribution of (A, B) Cu@NCS and, (C, D) Co/Cu@NCS.

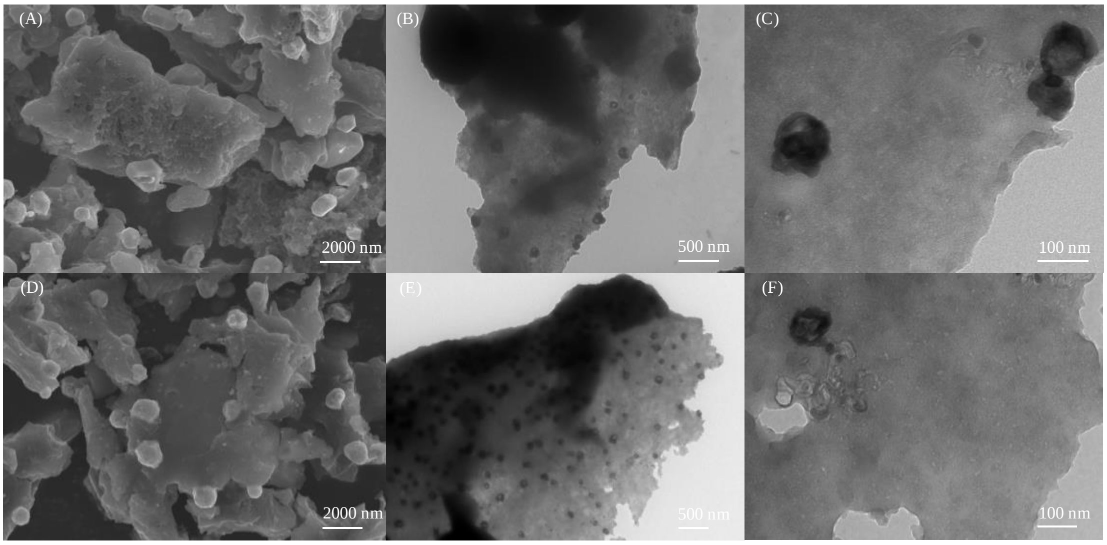

Figure S8. (A) SEM and (B, C) TEM images of Cu@NCS, and (D) SEM and (E, F) TEM images of Co/Cu@NCS.

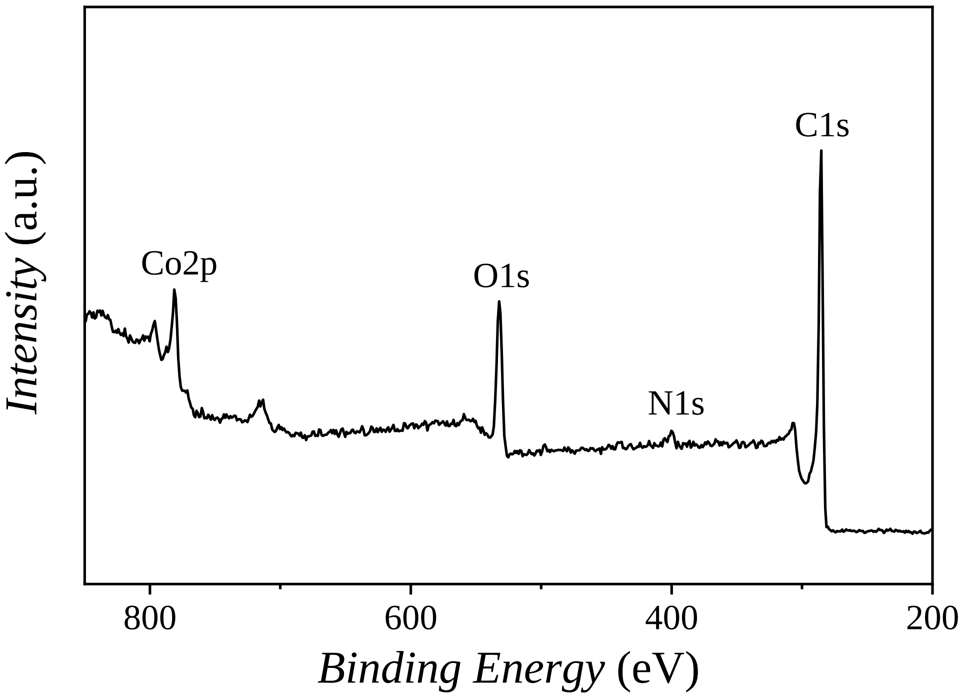

Figure S9. XPS survey of Co/CoO@NCS.

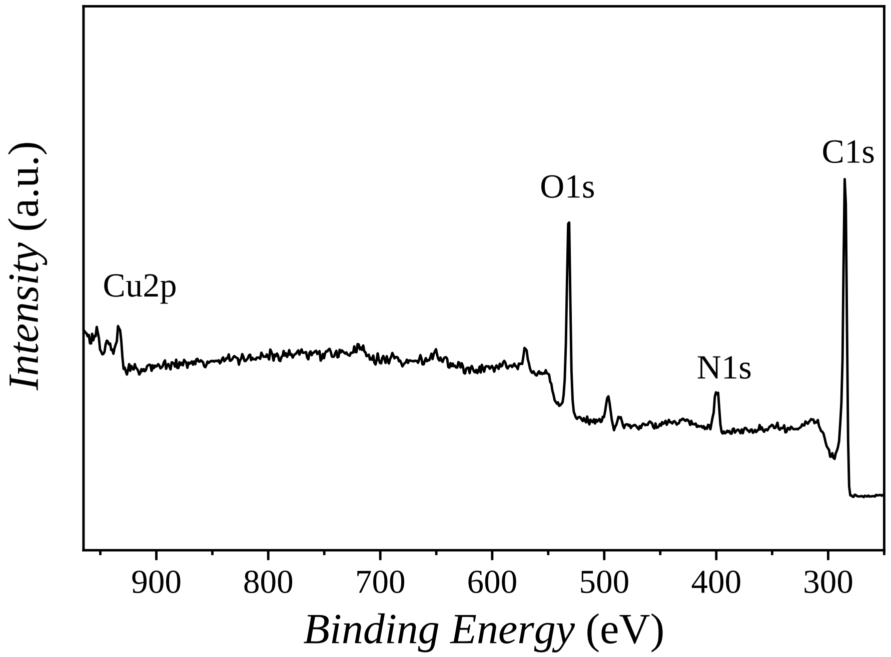

Figure S10. XPS survey of Cu@NCS.

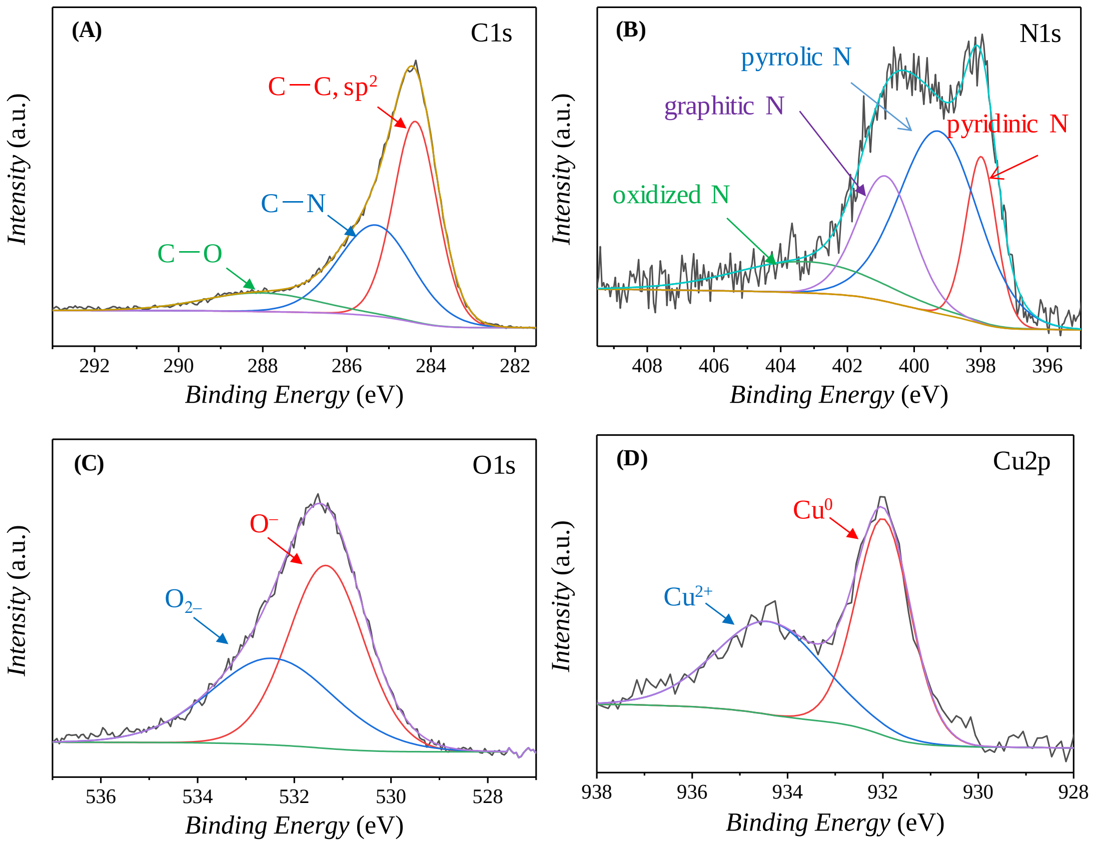

Figure S11. High resolution XPS spectra of Cu@NCS: (A) C1s, (B) N1s, (C) O1s and, (D) Cu2p.

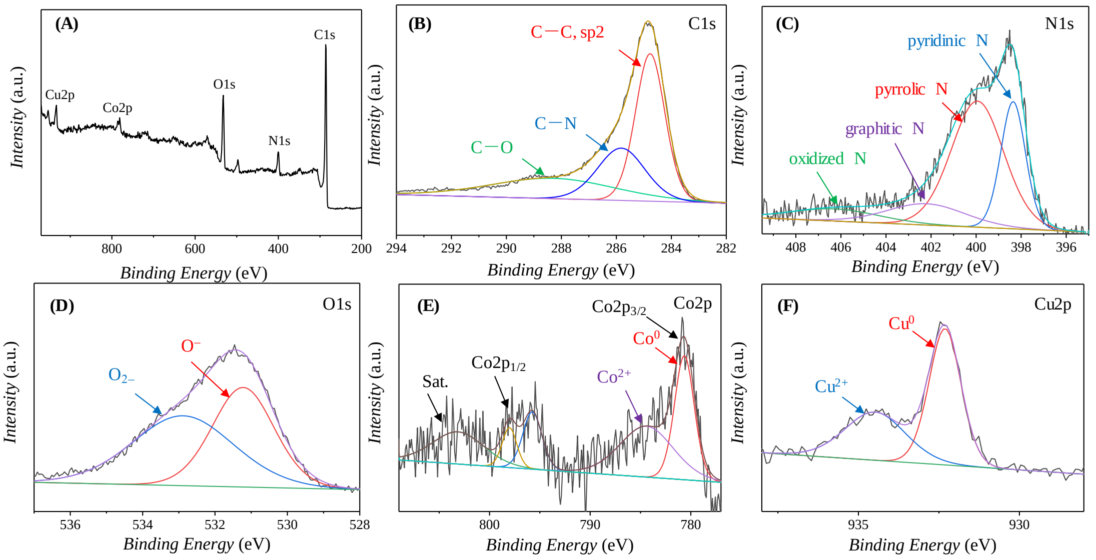

Figure S12. (A) XPS survey and (B-F) high resolution XPS spectra of Co/Cu@NCS: (B) C1s, (C) N1s, (D) O1s, (E) Co2p and (F) Cu2p.

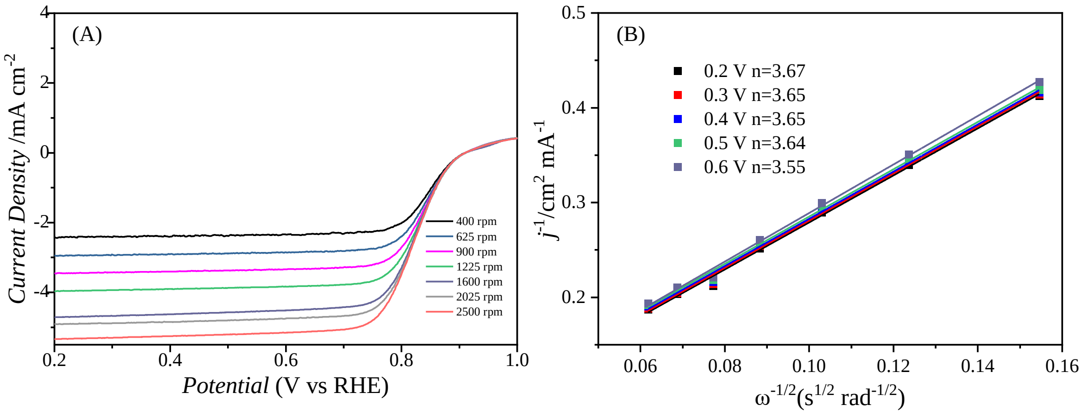

Figure S13. (A) LSV curves at different rotation speed and (B) K-L plots of Co/CoO@NCS at different potentials in O_2-saturated 0.1 M KOH.

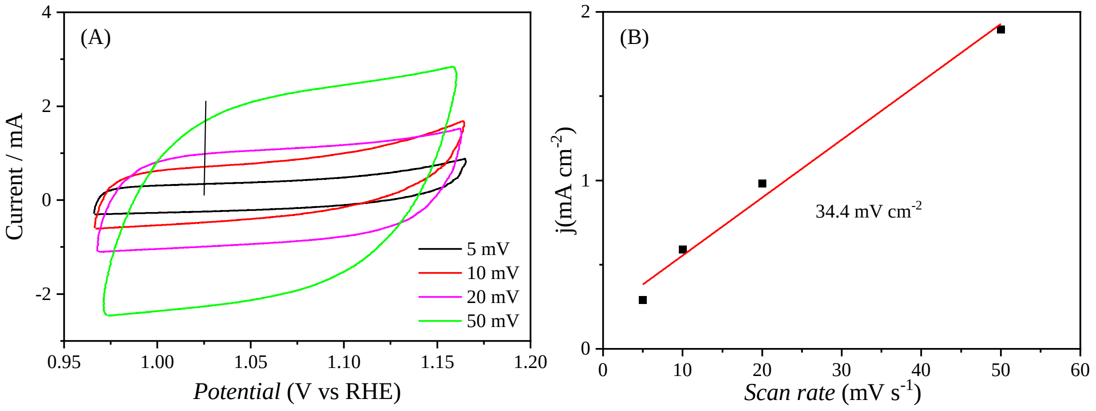

Figure S14. (A) CV curves of Co/CoO@NCS at different scan rate and (B) corresponding calculated electric double layer capacitance.

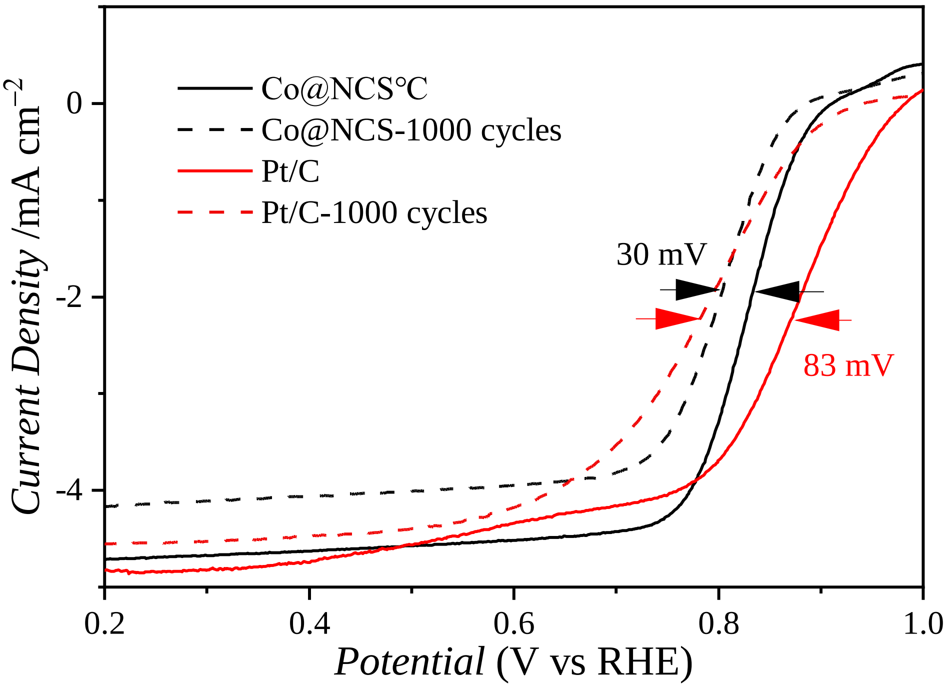

Figure S15. LSV curves of Co/CoO@NCS and Pt/C before and after 1000 cycles.
